# Methylpiperidinopyrazole Attenuates Estrogen-Induced Mitochondrial Energy Production and Subsequent Osteoblast Maturation via an Estrogen Receptor Alpha-Dependent Mechanism

**DOI:** 10.3390/molecules25122876

**Published:** 2020-06-22

**Authors:** Poh-Shiow Yeh, Jui-Tai Chen, Yih-Giun Cherng, Shun-Tai Yang, Yu-Ting Tai, Ruei-Ming Chen

**Affiliations:** 1 Department of Neurology, Chi Mei Medical Center, Tainan 710, Taiwan; poh.shiow@msa.hinet.net; 2 Graduate Institute of Medical Sciences, College of Medicine, Taipei Medical University, Taipei 110, Taiwan; 3 Department of Anesthesiology, School of Medicine, College of Medicine, Taipei Medical University, Taipei 110, Taiwan; cjuitai@tmu.edu.tw (J.-T.C.); stainless@s.tmu.edu.tw (Y.-G.C.); 4 Department of Anesthesiology, Shuang Ho Hospital, New Taipei City 235, Taiwan; 5 Department of Neurosurgery, School of Medicine, College of Medicine, Taipei Medical University, Taipei 110, Taiwan; 08512@s.tmu.edu.tw; 6 Cell Physiology and Molecular Image Research Center; Department of Anesthesiology, Wan Fang Hospital, Taipei Medical University, Taipei 116, Taiwan; 7 TMU Research Center of Cancer Translational Medicine, Taipei Medical University, Taipei 110, Taiwan; 8 Anesthesiology and Health Policy Research Center, Taipei Medical University, Taipei 110, Taiwan

**Keywords:** estrogen, methylpiperidinopyrazole, osteoblast maturation, energy production, osteogenesis

## Abstract

An estrogen deficiency is the main cause of osteoporosis in postmenopausal women. In bone remodeling, estrogen receptors (ERs) can mediate estrogen-transducing signals. Methylpiperidinopyrazole (MPP) is a highly specific antagonist of ER-alpha (ERα). This study was designed to evaluate the effects of MPP on estrogen-induced energy production, subsequent osteoblast maturation, and the possible mechanisms. Exposure of primary osteoblasts isolated from neonatal rat calvarias to MPP did not affect cell morphology or survival. Estradiol can induce translocation of ERα into mitochondria from the cytoplasm. Interestingly, pretreatment of rat calvarial osteoblasts with MPP lowered estrogen-induced ERα translocation. Sequentially, estrogen-triggered expressions of mitochondrial energy production-linked cytochrome c oxidase (COX) I and COX II messenger (m)RNAs were inhibited following pretreatment with MPP. Consequently, MPP caused decreases in estrogen-triggered augmentation of the activities of mitochondrial respiratory complex enzymes and levels of cellular adenosine phosphate (ATP). During progression of osteoblast maturation, estrogen induced bone morphogenetic protein (BMP)-6 and type I collagen mRNA expressions, but MPP treatment inhibited such induction. Consequently, estrogen-induced osteoblast activation and mineralization were attenuated after exposure to MPP. Taken together, MPP suppressed estrogen-induced osteoblast maturation through decreasing chromosomal osteogenesis-related BMP-6 and type I collagen mRNA expressions and mitochondrial ATP synthesis due to inhibiting energy production-linked COX I and II mRNA expressions. MPP can appropriately be applied to evaluate estrogen-involved bioenergetics and osteoblast maturation.

## 1. Introduction

Osteoporosis, a chronic and age-related bone disorder, occurs because of continual loss of bone mass [1]. The quality of bone is maintained through bone remodeling, a dynamic process that is a balance between osteoblast-mediated bone formation and osteoclast-involved bone resorption [2]. An imbalance of bone remodeling can lead to bone diseases such as osteoporosis, osteopenia, and Paget’s disease. Both women and men over the age of 50 years may suffer from osteoporosis at 1:3 and 1:5 ratios, respectively [3]. After women undergo menopause, levels of serum estrogen, the primary female sex hormone, dramatically drop, and an estrogen deficiency is a major cause of osteoporosis in postmenopausal women [4]. As to the mechanisms, a reduction in levels of serum estrogen is frequently associated with elevated activities of osteoclasts, resulting in faster bone resorption than formation and ultimate loss of bone mass [5]. Estrogen receptors (ERs), including the alpha (α) and beta (β) isoforms, mediate estrogen-transducing signals to regulate osteogenesis- and osteoclastogenesis-linked gene expressions through specific binding to estrogen response elements (EREs) [6]. Thus, estrogen plays critical roles in maintaining bone remodeling and bone mass.

Fragility fractures are a chief complication in osteoporotic patients [7]. Combined with additional chronic diseases, osteoporosis-associated bone breakage may cause higher risks of morbidity and mortality [8]. In comparison, it is much more difficult to recover from osteoporosis-linked fractures than from common broken bones. Hence, patients with an osteoporotic fracture usually experience long-term disability and a lower quality of life [8]. Simultaneously, an osteoporotic fracture creates economic burdens to families and nations worldwide. Osteogenesis, the progression of osteoblast maturation involves proliferation and differentiation of osteoprogenitor cells, extracellular matrix production and mineralization, osteocyte formation, and calcification, and contributes to bone formation and fracture healing [9]. Estrogen can preserve bone homeostasis via stimulating osteogenesis [10]. In female mice, activation of the estrogen-ERα axis is essential for expansion of cortical and trabecular bone [11]. Osteoblasts, differentiated from mesenchymal stem cells, directly participate in osteogenesis, bone formation, and fracture repair [12]. Numerous factors are involved in osteoblast maturation [13]. Increased expression of alkaline phosphatase (ALP), a characteristic biomarker of osteoblasts, is associated with progressive differentiation of osteoblasts [14,15]. Moreover, type I collagen and bone morphogenetic protein (BMP)-6 are two vital proteins that contribute to osteoblast differentiation and mineralization [13,15,16]. Clarifying expression levels of these gene is important for evaluating osteoblast maturation.

Osteogenesis and fracture healing require energy. Adenosine triphosphate (ATP), an energy-carrying molecule, is synthesized in mitochondria via respiratory chain reactions [17]. Mitochondrial respiratory chain complexes, including complexes I~V, carry out these electron transport reactions and subsequent ATP synthesis. In the mitochondrial respiratory chain, cytochrome c oxidases (COXs), referred to as complex IV, are unique terminal oxidases constructed of 13 subunits that catalyze the transfer of electrons from cytochrome c to oxygen [18]. Among these 13 subunits, COX I and II are encoded in mitochondrial DNA. Our previous study demonstrated that estradiol can induce mitochondrial *COX I* gene expression and then stimulate ATP synthesis in osteoblasts via activating ERα [19]. Estrogen plays important roles in bone remodeling and fracture healing via activation of ERα [6]. Moreover, estrogen deficiency is a major cause for the incidence of osteoporosis [20]. In spite of hormone replacement treatment (HRT) being reported to be associated with decreased risks of osteoporosis-associated fracture, HRT may increase risks of cardiovascular diseases, venous thromboembolisms, and breast cancer [21]. Instead, phytoestrogens, plant-derived xenoestrogens, were reported to have potential for treating bone disorders [22,23]. For example, genistein, a phytoestrogen, stimulated osteoblast maturation via an ERα-dependent pathway [24]. In an orchidectomized rat model of osteoporosis, genistein could prevent bone loss [25]. Methylpiperidinopyrazole (MPP) is a nonsteroidal and highly specific antagonist of ERα [26]. Compared to other ERα antagonists, MPP has the best specificity to suppress ERα activation. Previous studies used MPP to show the roles of estrogen in ovulation in rats and host immunity in response to a urinary tract infection [27,28]. In contrast, the action of MPP on estrogen-induced osteogenesis is known little. Therefore, this study isolated primary rat osteoblasts as our experimental model to further evaluate the effects of MPP on estrogen-induced osteoblast maturation and the possible mechanisms.

## 2. Results

### 2.1. MPP Did Not Induce Cytotoxicity in Rat Calvarial Osteoblasts

**Figure 1:**
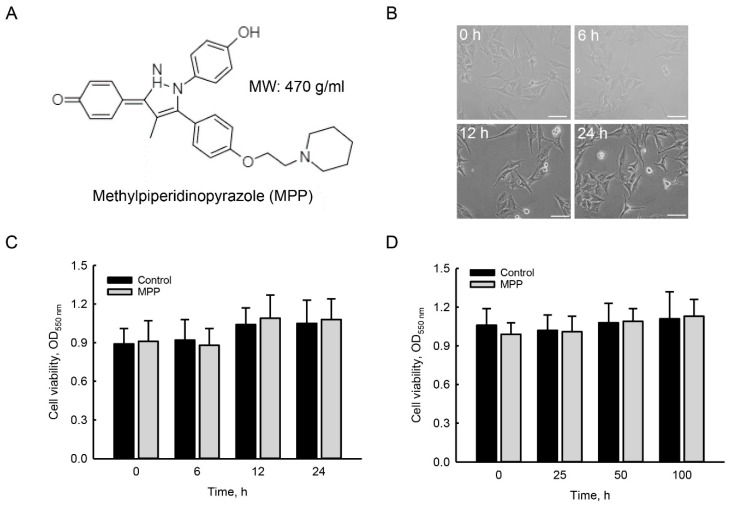
Methylpiperidinopyrazole (MPP) did not affect the morphology or survival of rat calvarial osteoblasts. The chemical structure and molecular weight (MW) of MPP are shown (**A**). Primary osteoblasts isolated from neonatal rat calvarias were exposed to 100 μM MPP for 6, 12, and 24 h. Cell morphology was observed and photographed using an inverted light microscope (**B**). In addition, rat calvarial osteoblasts were treated with 100 μM MPP for 6, 12, and 24 h or with 25, 50, and 100 μM MPP for 24 h. Survival of primary rat osteoblasts was analyzed using a trypan blue exclusion method (**C**,**D**). Each value represents the mean ± standard deviation of at least three independent determinations. Scale bar: 50 μM.

Effects of MPP on cytotoxicity toward rat calvarial osteoblasts were determined by analyzing cell morphology and survival (Figure 1). The chemical structure of MPP is shown in Figure 1A. The molecular weight of MPP is 470 g per mole. Exposure of primary rat osteoblasts to 100 μM MPP for 6 h did not change the cell morphology or numbers (Figure 1B). When the treatment period was extended to 12 and 24 h, morphologies and numbers of rat calvarial osteoblasts were still not altered by MPP. In parallel, treatment of rat calvarial osteoblasts with 100 μM MPP for 6 h did not affect cell survival compared to the control group (Figure 1C). At 12 and 24 h of treatment, survival of primary rat osteoblasts was not influenced following exposure to 100 μM MPP. Compared to untreated rat osteoblasts, treatment with 25, 50, and 100 μM MPP for 24 h did not change cell survival (Figure 1D).

### 2.2. MPP Diminished Estradiol-Induced Translocation of ERα from the Cytoplasm To Mitochondria in Rat Calvarial Osteoblasts

**Figure 2:**
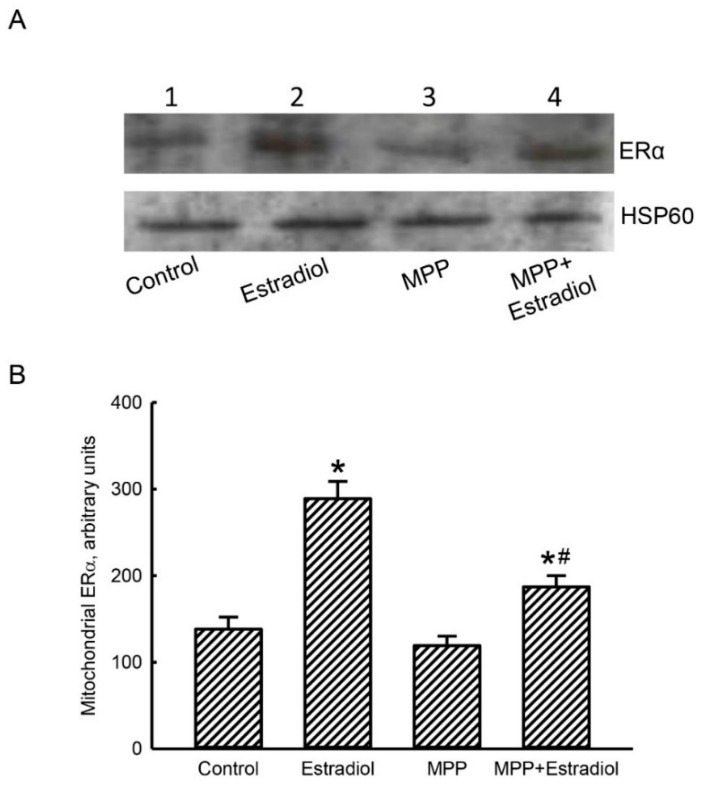
Methylpiperidinopyrazole (MPP) decreased estradiol-induced translocation of estrogen receptor-α (ERα) to mitochondria from the cytoplasm in rat calvarial osteoblasts. Primary osteoblasts isolated from neonatal rat calvarias were exposed to 10 nM estradiol, 100 μM MPP, and a combination of MPP and estradiol for 24 h. After treatment, mitochondrial proteins were prepared, and their concentrations were measured. These mitochondrial proteins were loaded onto sodium dodecylsulfate (SDS)-polyacrylamide gel electrophoresis (PAGE) followed by electronic separation. After being electronically transferred onto membranes, levels of mitochondrial ERα were immunodetected (**A**, top panel). Amounts of heat shock protein 60 (HSP60) in membranes were measured as the internal control for mitochondrial protein loading (bottom panel). These immune-related protein bands were quantified using HSP60 as a loading control, and these data were statistically analyzed (**B**). Each value represents the mean ± standard deviation of at least three independent determinations. The symbols * and ^#^ indicate that values significantly (*p* < 0.05) differed from the control and estradiol-treated groups, respectively.

An immunoblot assay was carried out to determine the effects of MPP and estradiol on levels of mitochondrial ERα in rat calvarial osteoblasts (Figure 2). In untreated osteoblasts, basal levels of mitochondrial ERα were detected (Figure 2A, top panel, lane 1). After exposure to 10 nM estradiol for 24 h, levels of mitochondrial ERα in rat calvarial osteoblasts were augmented (lane 2). Pretreatment of primary rat osteoblasts with 100 μM MPP alone did not influence amounts of mitochondrial ERα (lane 3). In contrast, a combined treatment of rat osteoblasts with MPP and estradiol caused a significant reduction in levels of mitochondrial ERα compared to the estradiol-treated group (lanes 2 and 4). Amounts of heat shock protein 60 (HSP60) in osteoblasts were immunodetected as the internal control of mitochondrial protein loading (bottom panel). These immune-related protein bands were quantified, normalized using HSP60 as the loading control, and statistically analyzed (Figure 2B). Exposure of rat calvarial osteoblasts to estradiol led to a significant 2.1-fold increase in levels of mitochondrial ERα. Pretreatment of primary rat osteoblasts with MPP alone did not change levels of mitochondrial ERα but caused a 68% attenuation in the estradiol-triggered translocation of ERα from the cytoplasm to mitochondria (Figure 2B).

### 2.3. MPP Concurrently Inhibited Estradiol-Induced Expressions of Mitochondrial Energy Production-Linked COX I and COX II mRNAs in Rat Calvarial Osteoblasts

**Figure 3:**
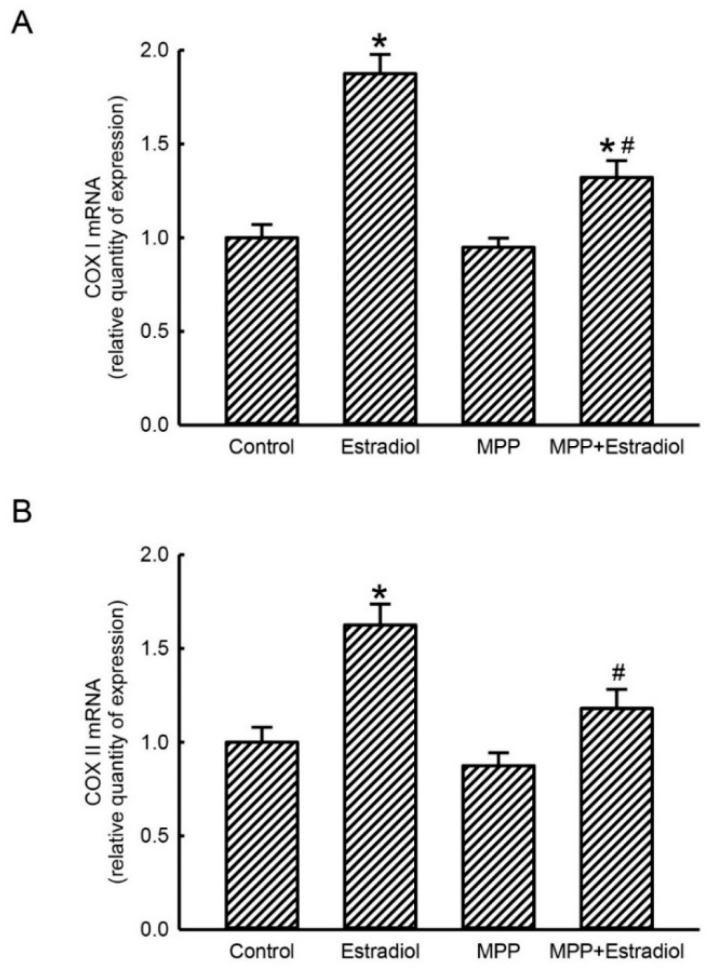
Methylpiperidinopyrazole (MPP) inhibited estradiol-induced expressions of mitochondrial cytochrome c oxidase (COX) I and II mRNAs in rat calvarial osteoblasts. Primary osteoblasts isolated from neonatal rat calvarias were exposed to 10 nM estradiol, 100 μM MPP, and a combination of MPP and estradiol for 24 h. After drug treatment, total RNAs were prepared from control and drug-treated primary rat osteoblasts. A real-time PCR analysis was further conducted to determine the effects of MPP and estradiol on expressions of mitochondrial COX I mRNA (**A**) and COX II mRNA (**B**). Each value represents the mean ± standard deviation of at least three independent determinations. The symbols * and ^#^ indicate that values significantly (*p* < 0.05) differed from control and estradiol-treated osteoblasts, respectively.

In parallel with the suppressive effects of MPP on estradiol-triggered translocation of ERα into mitochondria from the cytoplasm, a real-time PCR analysis was further carried out to determine the actions of MPP and estradiol on mitochondrial ATP synthesis-associated COX I and II mRNA expressions (Figure 3). Treatment of rat calvarial osteoblasts with 10 nM estradiol for 24 h caused a 2.2-fold induction of mitochondrial COX I mRNA expression (Figure 3A). Pretreatment with MPP alone did not influence basal levels of mitochondrial COX I mRNA in primary rat osteoblast. Nevertheless, the estradiol-induced enhancement of the expression of mitochondrial COX I mRNA in rat osteoblasts was significantly inhibited by 73% following pretreatment with MPP (Figure 3A). Compared to the control group, treatment of rat calvarial osteoblasts with 10 nM estradiol for 24 h led to a noteworthy 63% increase in mitochondrial COX II mRNA expression (Figure 3B). Levels of mitochondrial COX II mRNA in primary rat osteoblasts were not affected by pretreatment with MPP. In contrast, pretreatment of rat calvarial osteoblasts with MPP caused comprehensive inhibition of the estradiol-induced increase in mitochondrial COX II mRNA expression (Figure 3B).

### 2.4. MPP Subsequently Lowered Estradiol-Triggered Enhancements of Mitochondrial Complex Enzyme Activities and Cellular ATP Levels in Rat Calvarial Osteoblasts

**Figure 4:**
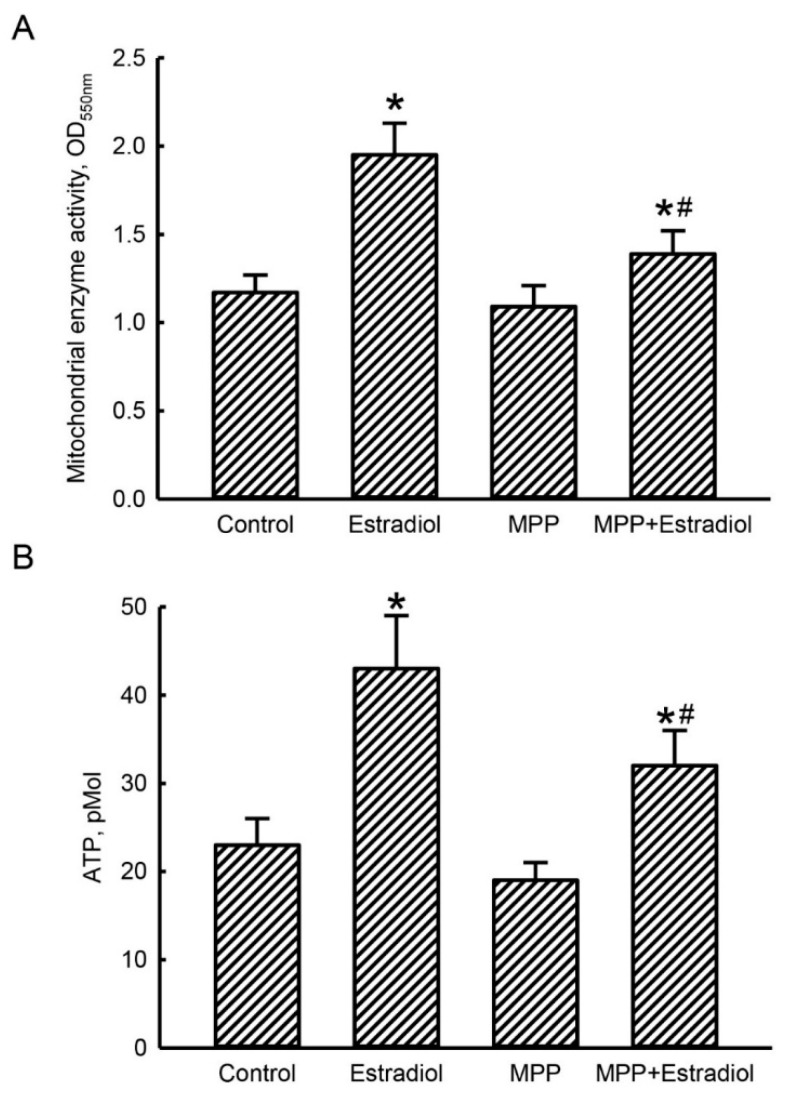
Methylpiperidinopyrazole (MPP) reduced estradiol-triggered enhancements in activities of mitochondrial respiratory complex enzymes and levels of cellular ATP in rat calvarial osteoblasts. Primary osteoblasts isolated from neonatal rat calvarias were exposed to 10 nM estradiol, 100 μM MPP, and a combination of MPP and estradiol for 24 h. After drug treatment, mitochondrial complex enzyme activities were assayed with a colorimetric method (**A**). Levels of cellular ATP were measured with a bioluminescence assay (**B**). Each value represents the mean ± standard deviation of at least three independent determinations. The symbols * and ^#^ indicate that values significantly (*p* < 0.05) differed from the control and estradiol-treated groups, respectively.

To determine successive actions of MPP-induced inhibition of mitochondrial energy production-associated COX I and II mRNA expressions in rat calvarial osteoblasts, mitochondrial respiratory complex enzyme activities and cellular ATP levels were examined (Figure 4). Exposure of rat calvarial osteoblasts to 10 nM estradiol for 24 h caused a 67% elevation in activities of mitochondrial NAD(P)H-dependent cellular oxidoreductase enzymes (Figure 4A). Pretreatment with MPP alone did not influence activation of mitochondrial complex enzymes in primary rat osteoblasts. However, estradiol-induced increases in activities of mitochondrial respiratory complex enzymes were repressed by 72% after pretreatment with MPP (Figure 4A).

In parallel with elevated activities of mitochondrial complex enzymes, treatment of rat calvarial osteoblasts with 10 nM estradiol for 24 h led to an 86% augmentation in levels of cellular ATP (Figure 4B). Synthesis of cellular ATP in primary rat osteoblasts was not changed by MPP pretreatment. Fascinatingly, treatment of rat calvarial osteoblasts with MPP meaningfully lowered the estradiol-induced enhancement of the synthesis of cellular ATP by 56% (Figure 4B).

### 2.5. MPP Accordingly Inhibited Estradiol-Induced Expressions of Osteoblast Maturation-Associated BMP-6 and Type I Collagen mRNAs in Rat Calvarial Osteoblasts

**Figure 5:**
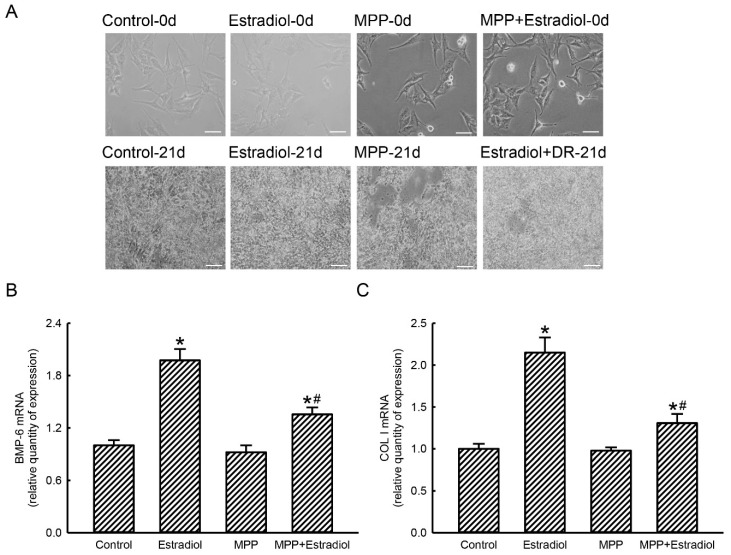
Methylpiperidinopyrazole (MPP) inhibited estradiol-induced expressions of osteoblast maturation-associated bone morphometric protein (BMP)-6 and type I collagen (COL I) mRNA expressions in rat calvarial osteoblasts. Primary osteoblasts isolated from neonatal rat calvarias were exposed to a differentiation agent with estradiol or with a combination of MPP and estradiol for 21 days. Control cells received dimethyl sulfoxide (DMSO) or MPP only. The differentiation agent, MPP, and estradiol were renewed every 2 days. Cell morphologies were observed and photographed using an inverted light microscope (**A**). After drug treatment, total RNAs were prepared from various groups using a TRIzol protocol. Levels of BMP-6 mRNA (**B**) and COL I mRNA (**C**) were analyzed using a real-time PCR. Amounts of β-actin mRNA were measured as the internal control. Each value represents the mean ± standard deviation of at least three independent determinations. The symbols * and ^#^ indicate that values significantly (*p* < 0.05) differed from the control and estradiol-treated groups, respectively. Scale bar: 50 μM.

The effects of MPP on estradiol-induced expressions of osteoblast maturation-linked *BMP-6* and *type I collagen* genes were next investigated (Figure 5). In the control group after being cultured for 21 days, compact morphologies of rat calvarial osteoblasts were observed (Figure 5A, left panels). Compared to untreated bone cells, treatment of rat calvarial osteoblasts with a differentiation reagent for 21 days did not change cell morphologies (middle panels). Exposure of primary rat osteoblasts to MPP alone or to a combination of MPP, estradiol, and a differentiation reagent for 21 days did not influence cell morphology (right two panels). Exposure of rat calvarial osteoblasts to a combination of estradiol and a differentiation reagent for 21 days induced expression of BMP-6 mRNA by 98% (Figure 5B). Levels of BMP-6 mRNA in rat calvarial osteoblasts were not altered after exposure to only MPP. In contrast, treatment of primary rat osteoblasts with MPP for 21 days caused a 63% inhibition in estradiol-induced BMP-6 mRNA expression (Figure 5B). Compared to the control group, combined treatment of rat calvarial osteoblasts with estradiol and a differentiation reagent for 21 days triggered 2.2-fold expression of type I collagen mRNA (Figure 5C). Treatment with MPP alone for 21 days did not affect collagen type I mRNA expression in rat calvarial osteoblasts. Nonetheless, exposure to MPP led to a significant 70% inhibition in estradiol-induced augmentation of type I collagen mRNA expression in rat calvarial osteoblasts (Figure 5C)

### 2.6. MPP Lowered Estradiol-Induced Activation and Mineralization of Rat Calvarial Osteoblasts

**Figure 6:**
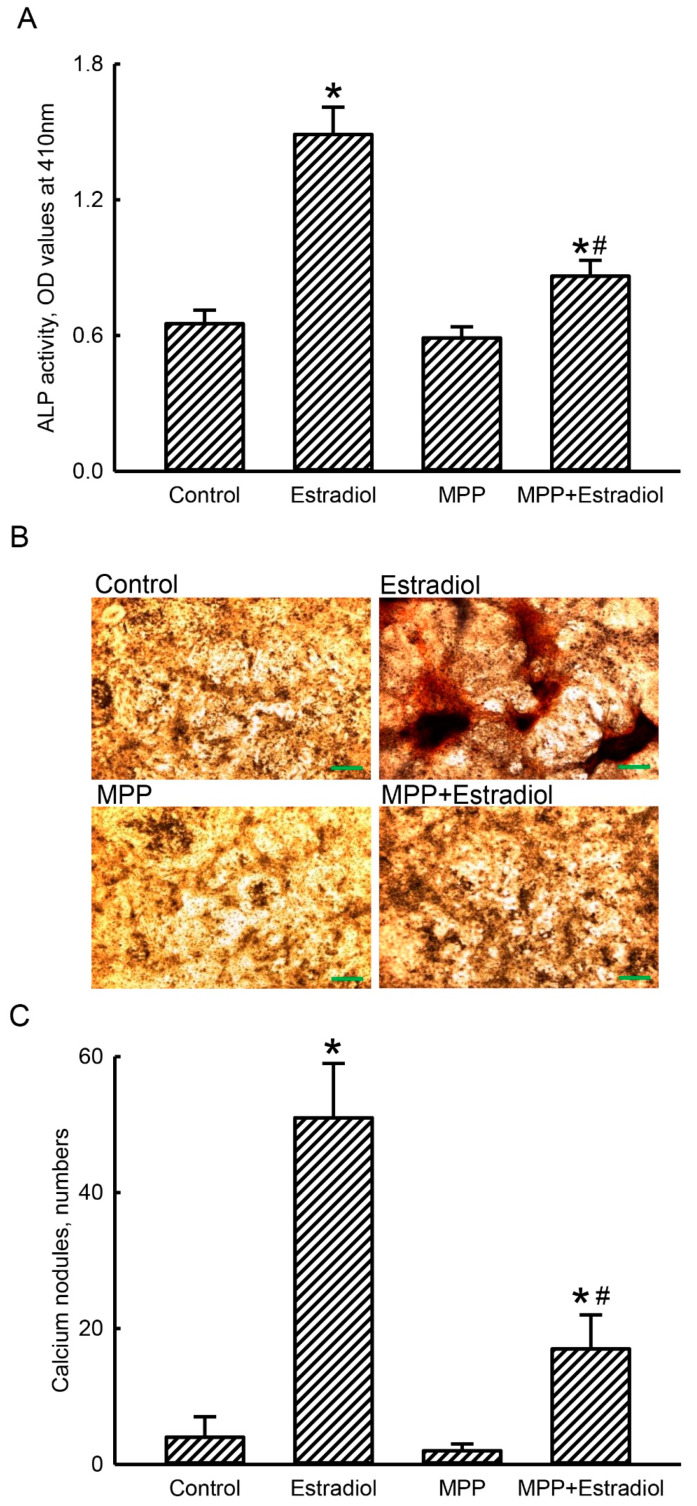
Methylpiperidinopyrazole (MPP) attenuated estradiol-induced activation and mineralization of rat calvarial osteoblasts. Primary osteoblasts isolated from neonatal rat calvarias were exposed to a differentiation agent with estradiol or with a combination of MPP and estradiol for 21 days. Control cells received DMSO or MPP only. The differentiation agent, MPP, and estradiol were renewed every 2 days. After drug treatment, activities of alkaline phosphatase (ALP) in rat calvarial osteoblasts were assayed using a colorimetric method (**A**). Osteoblast mineralization was examined using an alizarin red S-staining protocol (**B**). Numbers of mineralized calcium nodules were quantified, and data were statistically analyzed (**C**). Each value represents the mean ± standard deviation of at least three independent determinations. The symbols * and ^#^ indicate that values significantly (*p* < 0.05) differed from the control and estradiol-treated groups, respectively. Scale bar: 200 μM.

ALP activity and cell mineralization were also evaluated to determine the effects of MPP on estradiol-induced activation and maturation of rat calvarial osteoblasts (Figure 6). Compared to the control group, exposure of rat calvarial osteoblasts to a combination of estradiol and a differentiation reagent for 21 days increased activity of ALP by 2.3-fold (Figure 6A). ALP activity in primary rat osteoblasts did not change after treatment with MPP alone. Administration of MPP led to a significant 75% inhibition in the estradiol-induced improvement of ALP activities in rat calvarial osteoblasts (Figure 6A).

In the control group, our imaging results by an alizarin red S-staining analysis showed that very few mineralized nodules had formed (Figure 5B, left-top panel). After combined treatment with estradiol and a differentiation reagent for 21 days, rat calvarial osteoblasts obviously produced mineralized nodules (right-top panel). Similar to the control group, treatment of rat calvarial osteoblasts with MPP produced very rare mineralization (left-bottom panel). Interestingly, exposure of primary rat osteoblasts to MPP perceptibly suppressed the estradiol-induced growth of calcium-mineralized nodules (right-bottom panel). These mineralized nodules were counted, and the data were statistically analyzed (Figure 5C). Treatment of rat calvarial osteoblasts with a combination of estradiol and a differentiation reagent for 21 days caused a significant 13-fold increase in the number of mineralized nodules. Compared to the control group, administration of MPP alone did not affect maturation of rat calvarial osteoblasts. However, exposure of rat calvarial osteoblasts to MPP led to a 67% reduction in the number of calcium-mineralized nodules (Figure 6C).

## 3. Discussion

Treatment with MPP suppressed estrogen-induced energy production and subsequent osteoblast maturation. The present study showed that exposure of rat calvarial osteoblasts to 100 μM MPP for 24 h caused no cytotoxicity. Under these administered conditions, estrogen-triggered augmentation of cellular ATP levels in rat osteoblasts was significantly attenuated after pretreatment with MPP. In osteogenesis, osteoblasts use glycolysis as the major metabolic pathway to produce ATP for cell differentiation [29]. Our present study also demonstrated the heightened effects of estrogen on activation of ALP in rat osteoblasts. ALP is a typical biomarker of osteoblasts [13,14]. Activation of the ALP enzyme corresponds to osteoblast maturation [15,30]. Moreover, after long-term treatment with estrogen, mineralization of rat calvarial osteoblasts was enhanced. Hence, estrogen can stimulate energy production and subsequent osteoblast maturation. In contrast, administration of MPP alleviated estrogen-induced ATP synthesis and osteoblast activation and maturation. Our previous study showed that knocking-down ERα using RNA interference concurrently repressed osteoblast mineralization [19]. MPP is a chief inhibitor of ERα [26]. Thus, MPP can reduce estrogen-triggered osteoblast maturation via an ERα-dependent pathway. An estrogen deficiency is a major cause of osteoporosis in postmenopausal women [4]. In addition, estrogen contributes to osteogenesis and bone formation [10]. Recently, phytoestrogens were reported to possess the potential to replace estrogen for treating bone disorders [22,23,24,31]. The present study showed the valuable actions of MPP for evaluating the effects of estrogen or phytoestrogens on osteoblast maturation, bone formation, and fracture healing.

Pretreatment of rat calvarial osteoblasts with MPP inhibited estrogen-induced mitochondrial COX I and II mRNA expressions via reducing translocation of ERα into mitochondria from the cytoplasm. Levels of mitochondrial ERα in rat osteoblasts were augmented following exposure to estrogen, but pretreatment with MPP diminished such translocation. ERα is a transcriptional factor that can bind to EREs to regulate chromosomal and mitochondrial ATP synthesis-associated gene expressions [6]. In parallel, estrogen-induced mitochondrial COX I and II mRNA expressions were inhibited by MPP. COX I and II are encoded in mitochondrial DNA [18]. Our previous study reported the existence of EREs in the 5′-promoter region of the *COX I* and COX *II* genes [19]. As a result, one possible reason explaining MPP-induced inhibition of estrogen-triggered mitochondrial COX I and II mRNA expressions is repression of ERα translocation from the cytoplasm to mitochondria. COX I and COX II are critical complex proteins in complex IV that can transfer electrons from cytochrome c to oxygen [18]. The estrogen-induced enhancement of cellular ATP levels in rat calvarial osteoblasts was attenuated after treatment with MPP. Enhancement in expression of mitochondrial COX I mRNA was associated with an improvement of ATP synthesis in osteoblasts [19]. In contrast, a mitochondrial COX deficiency may lead to ATP depletion and mitochondrial diseases [18]. Thus, MPP-induced inhibition of mitochondrial *cox I* and *II* genes in rat calvarial osteoblasts may lead to a reduction in ATP synthesis and consequent cell maturation.

MPP alleviated estrogen-induced expansion of ATP synthesis in rat calvarial osteoblasts. In parallel with inhibition of mitochondrial COX I and II mRNA expressions, MPP reduced estrogen-induced elevations in activities of mitochondrial respiratory complex enzymes. Mitochondrial respiratory chain complexes execute chain reactions of electron transportation to produce cellular ATP [29]. Estrogen can trigger mitochondrial DNA expression and respiratory complex enzyme activities via activation of the nuclear respiratory factor-1-mitochondrial transcription factor A pathway [32]. Subsequently, this study proved the suppressive effects of MPP on levels of cellular ATP in rat osteoblasts. In the brain of ovariectomized rats, administration of estrogen increased mitochondrial respiratory chain reactions and ATP synthesis coupled with increased expression and activity of complex IV [33]. Our previous study also reported the participation of the estrogen-ERα axis in ATP synthesis in osteoblasts through regulation of chromosomal and mitochondrial energy production-associated gene expressions [19]. Therefore, MPP-induced decrease in estradiol-induced increases in levels of cellular ATP in rat calvarial osteoblasts was mainly due to repression of *cox* gene expressions and mitochondrial respiratory complex enzyme activities.

MPP alleviated estrogen-induced osteoblast maturation via declining ATP production. In the progression of osteoporosis, induction of BMP-6 expression can concomitantly improve osteoblast maturation and bone formation [34]. Moreover, when culturing bone marrow cells in a matrix gel containing type I collagen, osteoblast differentiation was stimulated [35]. Our previous study showed the effects of genistein on induction of type I collagen mRNA expression in mouse MC3T3-E1 cells [24]. Also, differentiated osteoblasts possessed higher ALP activity and collagen synthesis. In the present study, we showed that estrogen-induced activation of ALP in rat calvarial osteoblasts and subsequent calcium mineralization were significantly attenuated following exposure to MPP. Thus, administration of MPP could inhibit estrogen-induced expressions of osteoblast mineralization-related *BMP-6* and *type I collagen* genes, thereby suppressing cell activation and maturation. A previous study used microinjection of MPP into rats to identify the roles of ERα in ovulation [27]. In response to a urinary tract infection, Sen et al. identified the involvement of ERα in modulation of host immunity in the bladder and kidneys using MPP to target this receptor [28]. Our present study showed improved roles of estrogen in enhancing energy production in osteoblasts and resulting cell mineralization via an ERα-dependent mechanism. This is the first study using MPP to verify the osteogenic effects of estrogen. Our previous study also demonstrated that genistein, a phytoestrogen in soybean, stimulates maturation of murine MC3T3-E1 cells through induction of *BMP-6* and *type I collagen* gene expressions [24]. Phytoestrogens were recently identified to have potential advantages for treating bone diseases [22,23]. As a result, MPP can be appropriately applied to evaluate the beneficial effects of estrogen and phytoestrogens on osteogenesis, bone formation, and fracture healing.

## 4. Materials and Methods

### 4.1. Preparation of Rat Osteoblasts

Primary osteoblasts were prepared from 3-day-old Wistar rat calvarias following a sequential enzymatic digestion method as described previously [36]. All procedures were performed according to the National Institutes of Health Guidelines for the Use of Laboratory Animals and were approved by the Institutional Animal Care and Use Committee of Taipei Medical University-Wan Fang Hospital, Taipei, Taiwan (Approval no. WAN-LAC-107-009). Briefly, newborn rats were sacrificed and their calvarias were collected. After removing adhering connective tissues, the calvarias were incubated with a sterile enzyme solution containing 0.1% collagenase, 0.05% trypsin, and 4 mM Na_2_EDTA in calcium- and magnesium-free phosphate-buffered saline (PBS, 0.14 M NaCl, 2.6 mM KCl, 8 mM Na_2_HPO4, and 1.5 mM KH_2_PO_4_) at room temperature for 20 min. This procedure was repeated to yield a total of six digests. After centrifugation, cells from each digestion were collected and seeded in Dulbecco’s modified Eagle’s medium (DMEM; Gibco-BRL, Grand Island, NY, USA) supplemented with 10% heat-inactivated fetal bovine serum (FBS), L-glutamine, penicillin (100 IU/mL), and streptomycin (100 μg/mL) in 10-cm tissue culture dishes at 37 °C in a humidified atmosphere of 5% CO_2_. Osteoblasts were grown to confluence prior to drug treatment. Only the first passage of rat osteoblasts was used in this study.

### 4.2. Drug Treatment

Rat calvarial osteoblasts were seeded onto tissue culture dishes for overnight incubation. MPP used in this study was purchased from Gibco-BRL. For each experiment, MPP was freshly dissolved in DMSO. Our preliminary data showed the safety of MPP at < 100 μM to rat calvarial osteoblasts. Primary rat osteoblasts were treated with 25, 50, or 100 μM MPP for 6, 12, or 24 h. Control osteoblasts received DMSO only. The concentrations of DMSO used in primary rat osteoblasts were all <0.1%. At this concentration, DMSO was not cytotoxic to rat calvarial osteoblasts. Estradiol was purchased from Sigma (St. Louis, MO, USA). Our previous study showed that estradiol at 10 nM could stimulate osteoblast maturation without affecting cell viability [19]. Thus, estradiol at 10 nM was used in this study.

### 4.3. Analyses of Cell Morphology and Cell Survival

Cytotoxicity of MPP to rat calvarial osteoblasts was determined by examining cell morphology and cell survival. Briefly, rat calvarial osteoblasts (2 × 10^4^ cells) were seeded in 24-well tissue culture plates overnight. Then, primary rat osteoblasts were treated with 25, 50, and 100 μM MPP for 24 h or 100 μM MPP for 6, 12, and 24 h. Morphologies of osteoblasts were observed and photographed using an inverted light microscope (Nikon, Tokyo, Japan) as described previously [37]. Cell survival was analyzed with a trypan blue exclusion method as described previously [38]. After treatment with MPP, rat calvarial osteoblasts were trypsinized with 0.1% trypsin-ethylenediaminetetraacetic acid (EDTA) (Gibco-BRL). Numbers of osteoblasts were counted under a light microscope and statistically analyzed.

### 4.4. Preparation of Mitochondrial Proteins

Mitochondrial proteins from control, MPP-, estradiol-, and MPP + estradiol-treated rat calvarial osteoblasts were prepared following a previously described method [39]. Briefly, rat calvarial osteoblasts (10^6^ cells) were seeded in 6-cm tissue culture dishes overnight. After drug treatment, rat osteoblasts were washed with PBS. Osteoblasts were harvested and homogenized in buffer A (250 mM sucrose, 20 mM 25 mM 4-(2-hydroxyethyl)-1-piperazineethanesulfonic acid (HEPES), 10 mM KCl, 1.5 mM MgCl_2_, 1 mM EDTA, 1 mM ethyleneglycoltetraacetic acid (EGTA), and 1 mM dithiothreitol). Following centrifugation, pellets were suspended in buffer B (1 mL buffer A containing 10 μL NP-40). After centrifugation, the supernatants were collected as the mitochondrial fractions.

### 4.5. Immunodetection of Mitochondrial ERα Protein

Levels of mitochondrial ERα from control, MPP-, estradiol-, and MPP + estradiol-treated rat calvarial osteoblasts were immunodetected as described previously [40]. When preparing mitochondrial proteins, a mixture of proteinase inhibitors, including 1 mM phenylmethylsulfonyl fluoride, 1 mM sodium orthovanadate, and 5 µg/mL leupeptin, was added to the buffer to prevent protein degradation by proteinases. Protein concentrations were quantified using a bicinchonic acid protein assay kit (Thermo, San Jose, CA, USA). Mitochondrial proteins (100 μg/well) were subjected to sodium dodecylsulfate (SDS)-polyacrylamide gel electrophoresis (PAGE). After that, proteins separated on the polyacrylamide gel were electronically transferred onto nitrocellulose membranes. After being blocked with 5% non-fat milk at 37 °C for 1 h, levels of ERα on the membrane were immunodetected using a rabbit polyclonal antibody (pAb) from Santa Cruz Biotechnology (Santa Cruz, CA, USA). HSP-60 was immunodetected as an internal control of mitochondrial proteins. Intensities of the immunoreactive protein bands were determined using a digital imaging system (Syngene, Cambridge, UK) and densitometry software (Syngene) as described previously [41].

### 4.6. Real-Time Polymerase Chain Reaction (PCR)

Expressions of mitochondrial COX I and COC II mRNAs and chromosomal BMP-6 and collagenase type I (COL I) mRNAs in rat calvarial osteoblasts were analyzed using a real-time PCR as described previously [42]. Rat calvarial osteoblasts (10^6^ cells) were seeded in 6-cm tissue culture dishes overnight. After exposure to MPP, estradiol, and a combination of MPP and estradiol, total RNAs from rat calvarial osteoblasts were isolated using the TRIzol reagent (Invitrogen, Carlsbad, CA, USA). mRNAs were then prepared for real-time PCR analyses of COX I, COX II, BMP-6, COL I, and β-actin mRNAs. Oligonucleotide primers were designed and synthesized by Mission Biotechnology (Taipei, Taiwan). Oligonucleotide sequences of the respective upstream and downstream primers were 5′-TACGTTGTAGCCCACTTCCACT-3′ and 5′-GGATAGGCCGAGAAAGTGTTGT-3′ for COX I mRNA; 5′-GTAGTACTCCCGATTGAAGCCC-3′ and 5′-ATTCTAGGACGATGGGCATGAA-3′ for COX II mRNA; 5′-CTTGTCCTCATGGCTGTGAAAC-3′ and 5′-TATTGCTGGTGCTCCTGGCTTC-3′ for COL I mRNA; 5′-AGGATGGGGTGTCAGAGGGAGA-3′ and 5′-GTTGTGCTGCGGTGTCACCA-3′ for BMP-6 mRNA; and 5′-ATGGATGATGATATCGCCGCGCTCGTCGTC-3′ and 5′-AGGGTGAGGATGCCTCTCTTGCTCTG-3′ for β-actin mRNA [19,24]. These mRNAs were reverse-transcribed into their complementary (c)DNAs. A real-time PCR analysis was carried out using iQSYBR Green Supermix (Bio-Rad, Hercules, CA, USA) and the MyiQ Single-Color Real-Time PCR Detection System (Bio-Rad). β-Actin mRNA was analyzed as the internal control. Real-time PCR data were quantified and statistically analyzed.

### 4.7. Assay of Mitochondrial Enzyme Activity

Activities of the mitochondrial NAD(P)H-dependent cellular oxidoreductase enzymes were assayed using a colorimetric method as described previously [43]. Briefly, rat calvarial osteoblasts (10^4^ cells) were seeded in 96-well tissue culture plates overnight. Following exposure to MPP, estradiol, and a combination of MPP and estradiol, primary rat osteoblasts were cultured with new medium containing 0.5 mg/mL 3-(4,5-dimethylthiazol-2-yl)-2,5-diphenyltetrazolium bromide (MTT) for a further 3 h. Blue formazan products in osteoblasts were dissolved in DMSO. Signals were then spectrophotometrically measured at a wavelength of 550 nm.

### 4.8. Measurement of Cellular ATP

Levels of cellular ATP in rat calvarial osteoblasts were analyzed using a bioluminescence assay according to the protocol for the Molecular Probes ATP Determination Kit (Molecular Probes, Eugene, OR, USA) as described previously [44]. The principle of this bioluminescence assay is based on the luciferase requirement for ATP in producing emitted light. Luminant light (560 nm) emitted by the luciferase-mediated reaction of ATP and luciferin was detected with a multilabel counter (Welch Allyn, Turku, Finland).

### 4.9. Examination of Osteoblast Mineralization

Mineralization of rat calvarial osteoblasts was examined using the alizarin red S dye-staining protocol as described previously [45]. Rat calvarial osteoblasts were seeded in 6-cm tissue culture dishes overnight. Osteoblasts were then treated with 100 μM MPP, a differentiation reagent (10 nM dexamethasone, 100 μg/mL ascorbic acid, and 10 mM β-glycerophosphate), a differentiation reagent + MPP, a differentiation reagent + estradiol, and a combination of a differentiation reagent + MPP + estradiol for 21 days. The differentiation reagent, MPP, and estradiol were renewed every 2 days. After drug treatment, the cell morphology was observed and photographed using a microscope (Nikon). Also, rat calvarial osteoblasts were washed with ice-cold PBS and then fixed in ice-cold 10% formalin for 20 min. For the alizarin red S dye protocol, fixed osteoblasts were thoroughly rinsed and then incubated in 1% alcian blue at pH 2.5 (ThermoFisher Scientific, Tewksbury, MA, USA) for 12 h. Sections were then incubated in alizarin red S (ThermoFisher Scientific) for 8 min, dehydrated briefly in xylene, and covered with a coverslip in Permount (ThermoFisher Scientific). Mineralized nodules were counted and statistically analyzed.

### 4.10. Analysis of ALP Activity

ALP activity in rat calvarial osteoblasts was assayed by a colorimetric method as described previously [46]. Primary rat osteoblasts were seeded overnight in 6-cm tissue culture dishes. Osteoblasts were then treated with 100 μM MPP, a differentiation reagent (10 nM dexamethasone, 100 μg/mL ascorbic acid, and 10 mM β-glycerophosphate), a differentiation reagent + MPP, a differentiation reagent + estradiol, and a combination of a differentiation reagent + MPP + estradiol for 21 days. The differentiation reagent, MPP, and estradiol were renewed every 2 days. After drug treatment, the ALP enzyme activity of primary rat osteoblasts was determined by detecting the formation of p-nitrophenol, a product of p-nitrophenyl phosphate catalyzed by ALP, according to a previously described colorimetric procedure (Sigma).

### 4.11. Statistical Analyses

The statistical significance of differences between groups was evaluated using a one-way analysis of variance (ANOVA) with Duncan’s multiple-range test. Differences were considered statistically significant at *p* values of < 0.05. Each value represents the mean ± standard deviation (SD).

## 5. Conclusions

This study successfully isolated rat calvarial osteoblasts as our experimental model to evaluate the effects of MPP on osteoblast maturation and the possible mechanisms. Exposure of rat calvarial osteoblasts to MPP did not lead to cytotoxicity but significantly lowered estradiol-induced translocation of ERα from the cytoplasm to mitochondria. Subsequently, the estradiol-induced mitochondrial COX I and II mRNA expressions, mitochondrial complex enzyme activation, and ATP synthesis were attenuated by MPP. Consequently, treatment of rat calvarial osteoblasts with MPP alleviated estradiol-induced osteoblast maturation-associated BMP-6 and type I collagen mRNA expressions, ALP activation, and cell mineralization. Therefore, MPP could suppress estradiol-triggered osteoblast activation and maturation through repressing chromosomal osteoblast maturation-associated gene expressions and mitochondrial energy production due to inhibition of ATP synthesis-linked gene expressions. MPP can be suitably applied for evaluation of estrogen- or phytoestrogen-induced osteoblast maturation, bone formation, and fracture healing. However, there are certain study limitations in this study. In the future, we will evaluate the effects of MPP on estrogen-induced alterations in levels of COX I, COX II, type I collagen, and BMP-6 proteins during osteoblast maturation.
